# Illusory interparty disagreement: Partisans agree on what hate speech to censor but do not know it

**DOI:** 10.1073/pnas.2402428121

**Published:** 2024-09-16

**Authors:** Brittany C. Solomon, Matthew E. K. Hall, Abigail Hemmen, James N. Druckman

**Affiliations:** ^a^Department of Management and Organization, University of Notre Dame, Notre Dame, IN; ^b^Department of Political Science, University of Notre Dame, Notre Dame, IN; ^c^Department of Political Science, University of Rochester, Rochester, NY

**Keywords:** hate speech, censorship, social media, misperceptions, conjoint survey experiment

## Abstract

Whether and when to censor hate speech are long-standing points of contention in the US. The latest iteration of these debates entails grappling with content regulation on social media in an age of intense partisan polarization. But do partisans disagree about what types of hate speech to censor on social media or do they merely differ on how much hate speech to censor? And do they understand out-party censorship preferences? We examine these questions in a nationally representative conjoint survey experiment (participant *N* = 3,357; decision *N* = 40,284). We find that, although Democrats support more censorship than Republicans, partisans generally agree on what types of hate speech are most deserving of censorship in terms of the speech’s target, source, and severity. Despite this substantial cross-party agreement, partisans mistakenly believe that members of the other party prioritize protecting different targets of hate speech. For example, a major disconnect between the two parties is that Democrats overestimate and Republicans underestimate the other party’s willingness to censor speech targeting Whites. We conclude that partisan differences on censoring hate speech are largely based on free speech values and misperceptions rather than identity-based social divisions.

## Significance

The ongoing violence in Israel that began in 2023 ignited antisemitic and anti-Palestinian speech in the United States, which intensified partisan debates about censoring hate speech on social media platforms. Given that hate speech reflects identity-based social divisions, censorship debates may stem from partisans’ propensity to protect groups with which they affiliate and consider vulnerable. If Democrats and Republicans tend to support censoring speech that targets marginalized groups and Whites, respectively, it would indicate a calcification of partisan-social group cleavages. However, we find substantial cross-party agreement on what hate speech should be censored-yet partisans mistakenly perceive disagreement. Debates on hate speech moderation should focus on understanding misperceptions of censorship preferences rather than what or who should be censored.

The outbreak of violence in Israel in late 2023 ignited an upsurge of antisemitic and anti-Palestinian speech in the United States, including dialogue on social media (1). The US Constitution protects most forms of hate speech (2)-that is, communication that promotes hatred, discrimination, or violence toward groups based on social identity markers. However, this constitutional guarantee does not leave hate speech entirely unencumbered. The government can regulate speech if it is deemed to incite lawlessness, pose a true threat, or breach the peace. Moreover, private entities, including social media platforms, can moderate online content as they see fit (3). Whether stemming from government regulation (responding to public preferences) or social media companies’ decisions (responding to market demands), censoring speech in society is a coordination problem. When speech is collectively deemed inappropriate, sanctions can be levied (4). But members of society must first establish what words are inappropriate in a given context via norms (5–7).

Debates about the parameters of (un)acceptable hate speech are far from new; however, agreeing on how to address hate speech in society faces two contemporary obstacles. First, the emergence of social media means that any speech, including hate speech, proliferates to public audiences very quickly and can be subject to third-party content moderation. Second, society has become increasingly polarized, both generally (8) as well as specifically regarding identity politics and censorship (e.g., cancel culture and book banning) (9–11). Thus, it remains unclear whether citizens can agree about censorship of hate speech on social media in this time of deep partisan division. Failure of partisans to coordinate on how to navigate hate speech makes communication more difficult, as some will employ inappropriate language that furthers social division while others will refrain from sharing appropriate ideas for fear of condemnation. Moreover, inconsistent expectations and behaviors by members of different parties could further fuel polarization and/or incentivize partisans to reside in distinct information ecosystems (12).

No work has directly addressed how partisan divisions manifest when it comes to hate speech censorship. We address this question by exploring two nonexclusive possibilities: 1) Partisans disagree about *what types* of speech should be censored (e.g., speech that targets certain groups, is posted by certain sources, or employs certain language), and/or 2) partisans disagree about *how much* censorship should occur (e.g., very little, a great deal) regardless of (dis)agreement on the types of speech that are censored. In other words, do Republicans and Democrats disagree about what hate speech to censor (based on identity politics), how much hate speech to censor (based on the desirability of free speech), or both?

Although disagreements over what to censor and how much to censor are nonexclusive, the difference between them is key. If partisans explicitly disagree on what types of hate speech to censor (and, thus, which specific groups deserve protection from hate speech), it suggests a further calcification of partisan-social group cleavages. However, partisans may instead simply disagree on the appropriate extent of censorship (and thus agree on the value and vulnerability of different social groups). Though we note that endorsing free speech values could still be problematic for society if it operates as a disguise for expressions of racism, as we consider in the discussion (13). Finally, regardless of what partisans actually prefer and what type of agreement may or may not exist, we also examine what partisans believe about the other side’s preferences. If partisans misperceive out-partisans as having more distinct censorship preferences than they do in reality, they may evade interparty communication for fear for eliciting out-party wrath, cling to ideological echo chambers, and become more distrusting of the out-party (14)-all despite actual agreement.

## Partisanship and What Hate Speech to Censor

We build on classic communication theories as our starting point for understanding censorship preferences by considering the speech’s target, source, and severity (15, 16). With regard to the target, partisans may disagree about which social groups deserve protection from hate speech. It is well documented that identity politics play an increasingly profound role in political polarization and thus shape American sociopolitical discourse and norms (9, 17). Given that partisanship and other social identities are psychologically intertwined (18), debates about hate speech censorship may be an especially straightforward iteration of partisan identity politics, pitting Democrats and marginalized groups against Republicans and socially dominant groups. A theoretically driven account of this dynamic stems from research indicating that partisan disagreement about issues related to harm, such as immigration policy and abortion, can be explained by fundamental differences in conceptions of victimhood based on assumptions of vulnerability (19). Liberals tend to emphasize concern for the “othered,” assuming that those who are outside the dominant social group are especially vulnerable to harm (20). And, indeed, Democrats generally strive to protect marginalized groups (e.g., racial and ethnic minorities) (13, 21, 22). Republicans, in contrast, not only seem to deny the victimization of the “othered” but are also apt to emphasize concern for the “powerful,” assuming that those in positions of power are susceptible to disadvantage (e.g., greater scrutiny, higher expectations, and exclusion due to their standing) (20). Republicans also tend to align more with social dominance orientation (23), driving their acceptance of minority-focused hate speech (24) and proneness to protect threats against Whites (9, 18, 25). Such disagreement about who is particularly vulnerable and warrants protection from harm should manifest in partisans’ censorship preferences, with Democrats supporting censorship of anti-Black, anti-Palestinian, and antisemitic speech (relative to censorship of anti-White speech), and Republicans supporting censorship of anti-White speech (relative to anti-Black or anti-Palestinian speech), all else constant (*hypothesis 1*). Given the timing of our study during the Israel–Hamas war, Republicans may prioritize censoring antisemitic speech relative to anti-White speech, and Democrats may prioritize restrictions on antisemitic as well as anti-Palestinian speech relative to anti-Black speech, all else constant (*corollary 1*).

The next key factor concerns the source of the speech. In prior work, the source’s party did not influence support for censorship of misinformation (7) or canceling speakers (12) on social media. However, the well-established literature on out-party animus and distrust (26) suggests that partisans will support censorship of out- versus in-party posts, and this dynamic may also emerge in the context of hate speech censorship. Accordingly, we expect that Democrats will be more likely to censor hate speech from Republicans (out-party) than from Democrats (in-party), and vice versa, all else constant (*hypothesis 2*). Apart from partisanship, we also investigate social positionality, focusing on private citizens, elected officials, and college professors. While some work suggests higher discursive standards for elected officials (27), the evolving normalization of uncivil elite rhetoric (28) raises the possibility that partisans do not necessarily believe that elected officials should be subject to greater expectations than ordinary citizens. We do predict that Republicans will be more supportive of censoring college professors (versus the average citizen), all else constant (*hypothesis 3*). This expectation reflects Republicans’ negative views of and concerns about indoctrination by professors (29, 30) and decreased confidence in higher education (30, 31). We examine the effect of professors as the source of posts due to long-standing discussions about free speech on college campuses (32, 33), although we recognize that doing so creates an asymmetry since respondents may associate professors with liberalism and we do not consider a stereotypically conservative source counterpart (e.g., a business executive).

Finally, we expect the severity of the hate speech content to matter. Prior research has found that censorship preferences are mainly driven by the perceived harm of the content for both Republicans and Democrats. For instance, support for censoring toxic social media posts is based primarily on the toxicity of the posts (34). And support for censoring right-wing misinformation is largely driven by the severity of the misinformation’s harm (3). In many contexts, incitement crosses legal boundaries, and dehumanization (while less proximately threatening) strongly correlates with violent inclinations (35, 36). Thus, partisans may be more likely to censor incitement than dehumanization than incivility than mere criticism, all else constant (*hypothesis 4*). That said, recent work suggests Republican (but not Democratic) elites and voters commonly invoke dehumanizing language (36–38). Such normalization suggests Republicans will not censor dehumanizing language any more than they censor uncivil language (*corollary 2*).

## Partisanship and How Much Hate Speech to Censor

Apart from what to censor, partisans may disagree about the appropriate amount of censorship (39–41). In recent years, there has been a realignment, such that Democrats now appear more censorial than Republicans (11, 21, 42). Thus, relative to Republicans, Democrats may exhibit more support for censorship of hate speech, all else constant (*hypothesis 5*). In this case, there could be cross-partisan agreement on what type of hate speech to censor but disagreement about how much to censor it. If so, censorship preferences would reflect free speech principles rather than identity-based partisan cleavages.

## Perceptions of Censoring Hate Speech

Regardless of whether partisans actually agree on what or how much hate speech to censor, partisans might misperceive out-party preferences, which could also result in ambiguous norms about what constitutes appropriate speech. Such misperceptions would likely exaggerate the same identity politics narrative that may generate actual disagreement. Think tanks (10, 43, 44) and the media (45, 46) often invoke identity-based partisan disagreements in discussing free speech, which can lead partisans to misperceive differences regardless of reality. This possibility aligns with work showing that partisans tend to exaggerate out-party stereotypes (47), out-party disagreement about in-party values and positions (48, 49), and political polarization (50). Along these lines, we expect Democrats to overestimate and Republicans to underestimate out-party support for censorship of hate speech targeting Whites, all else constant (*hypothesis 6*). And Democrats will underestimate and Republicans will overestimate out-party support for censorship of hate speech targeting marginalized groups (Blacks, Palestinian, and Jewish targets), all else constant (*hypothesis 7*).

## Experiment

**Fig. 1.**
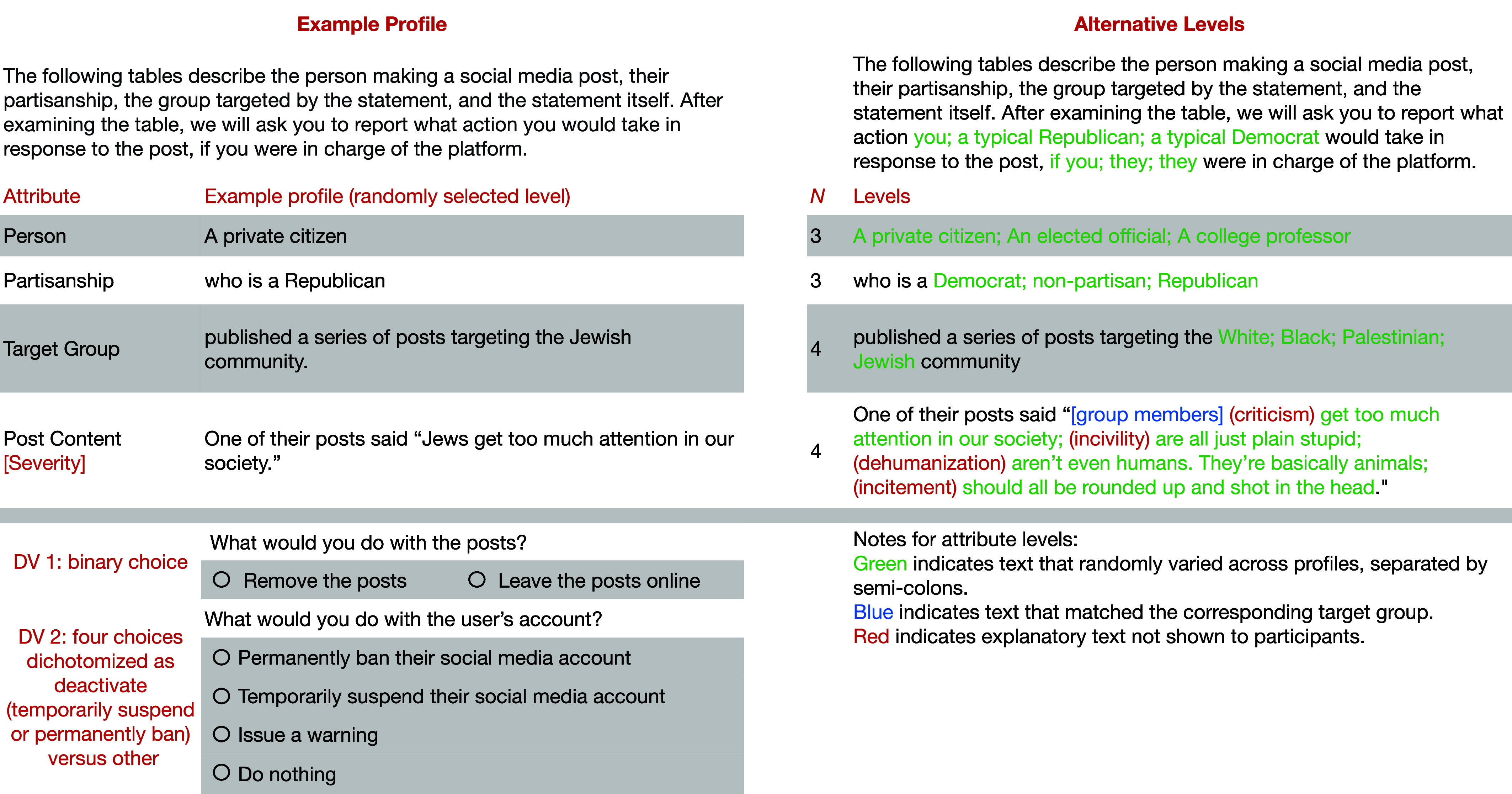
Example profile and conjoint profile design.

We used a single-profile conjoint survey experiment to test which factors influence willingness to censor hate speech on social media, as well as in-party and out-party perceptions of the typical partisan’s willingness to do so. A conjoint design is well suited for this research because various factors may influence these choices (51). Moreover, conjoint designs vitiate social desirability bias (52), and online survey experiments seem to be robust to experimenter demand effects (53). Each profile described a series of social media posts containing potentially objectionable speech targeting a particular racial/ethnic group with four randomly assigned attributes (in alignment with our hypotheses): the target group, the source’s partisanship, the source’s position in society, and the severity of the content (see Fig. 1 for an example). This design yielded 144 possible unique profiles. We recruited 3,357 participants via Forthright Access in December of 2023, quota-matched to the US general population. Participants were shown four profiles and asked whether they would remove the posts (“What would you do with the posts?”) and/or deactivate the user’s account (“What would you do with the user’s account?”) if they were in charge of the social media platform. Note that we do not use the term “censor” in our questions to reduce the possibility of partisan valence. Next, participants were shown eight variations of the profile and asked whether they think a typical Republican or Democrat (four in-party, then four out-party) would remove the posts and/or deactivate the user’s account if that person was in charge of the social media platform. (Below, we refer to both removal and deactivation as censorship.) The full experimental design and sample information are described in *Materials and Methods*.

## Results

### Censorship Decisions.

Participants chose to remove posts containing hate speech in the majority of profiles (aggregating across all attribute levels), regardless of the target group. Removal was most common for posts targeting Blacks (60.4%) and Jews (58.6%), but majorities also removed posts targeting Palestinians (54.8%) and Whites (54.6%). Fewer participants chose to deactivate accounts that posted hate speech, but more than 40% deactivated the account regardless of the target group. Again, deactivation was most common for posts targeting Blacks (50.8%) and Jews (47.7%). Although opposition to censorship was not a majority preference, we note that it was still fairly strong across target groups (roughly 40 to 45%), even when the speech targeted traditionally marginalized groups.

### Censorship Decisions by Political Party.

**Fig. 2.**
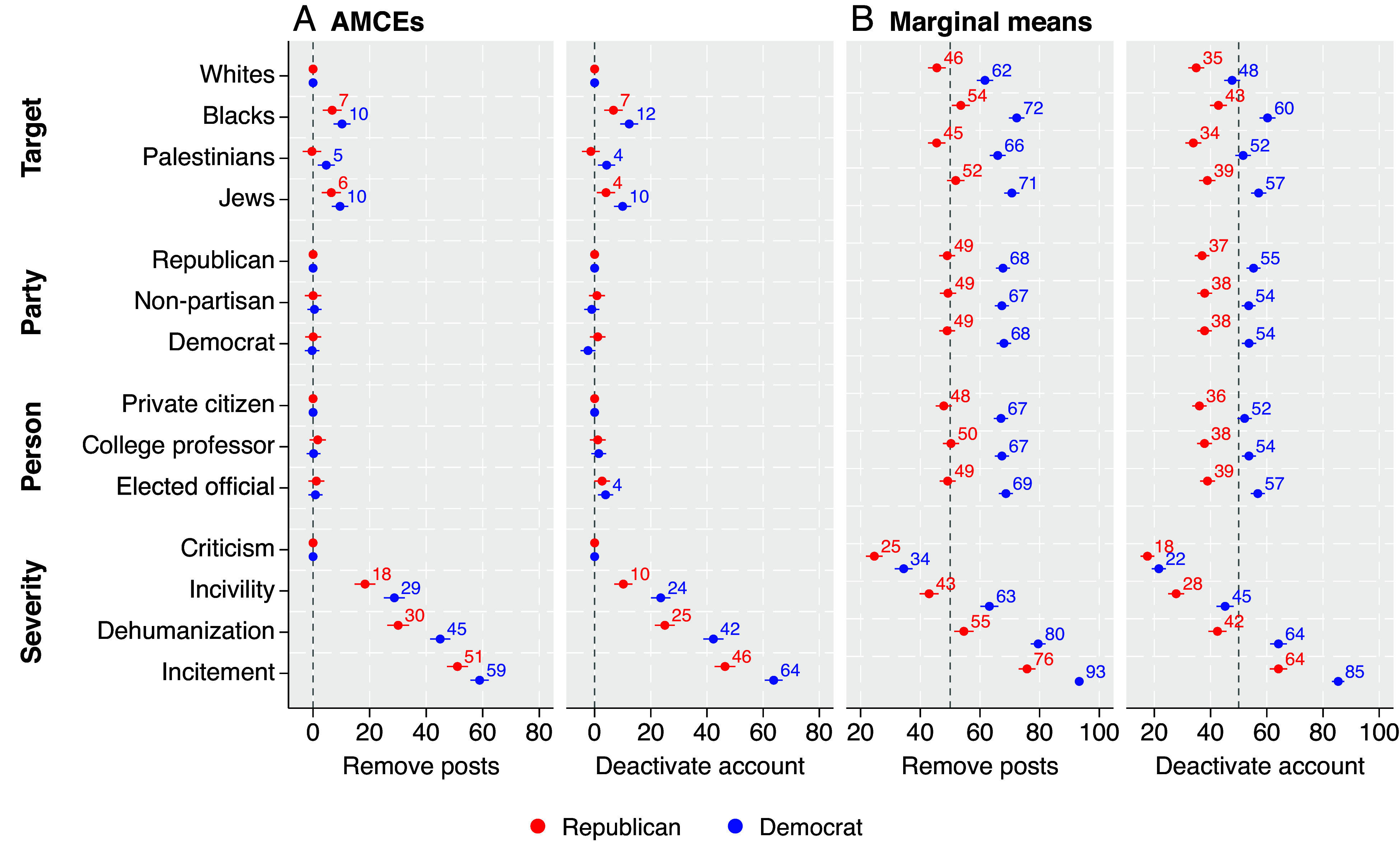
Figure reports (*A*) average marginal component effects (AMCEs) and (*B*) marginal means (MMs) of decisions to remove posts and deactivate accounts by party, plotted with 95% CIs. All values are percentage points.

Fig. 2*A* presents average marginal component effects (AMCEs) of each attribute level on removal and deactivation decisions compared to a baseline comparison level. (See *SI Appendix* for comparisons to independents.) Contrary to the expectation of disagreement (*hypothesis 1*), both Republicans and Democrats prioritized censoring hate speech that targets Blacks and Jews versus hate speech that targets Whites. (However, the AMCEs of targeting Blacks [*F* = 5.76, *P* = 0.02] and Jews [*F* = 6:80, *P* < 0:01] on deactivation were slightly stronger for Democrats than Republicans.) In short, partisans agreed on what types of speech to censor. The one notable difference was that Democrats, but not Republicans, were more likely to censor hate speech targeting Palestinians than hate speech targeting Whites (even though Republicans did not favor censoring anti-White hate speech per se). Otherwise, partisans agreed on which target groups to prioritize when censoring speech. Therefore, the results, at least partially, contradict both i and *corollary 1*. Regarding the former, Republicans do not prioritize censoring anti-White speech over anti-Black speech, and they are indifferent to censoring anti-Palestinian speech relative to anti-White speech. Regarding the latter, Democrats did not prioritize censoring antisemitic or anti-Palestinian speech relative to anti-Black speech.

Also contrary to the expectation of disagreement, neither the source’s partisanship (*hypothesis 2*) nor their position in society (i.e., the “person” attribute; *hypothesis 3*) affected censorship decisions, with one unexpected difference: Democrats were more likely to deactivate accounts owned by elected officials versus private citizens. However, partisans were not more supportive of censoring posts by out-party members, and Republicans were not more supportive of censoring posts by college professors. Thus, like the target results, partisans agreed on hate speech censorship based on the source—largely in that source does not matter.

We do, however, find evidence consistent with *hypothesis 4*, such that Republicans and Democrats were both more likely to censor posts as the severity of the hate speech (i.e., the “post content” attribute) increased. Language severity had stronger effects on Democrats than on Republicans. However, contrary to our expectation of difference here (*corollary 2*), Republicans (versus Democrats) were not more accepting of dehumanizing language (relative to incivility). Thus, partisans agreed on censoring hate speech based on the severity of the language. Taken together, partisans generally agree on what to censor when it comes to the target, source, and severity of hate speech.

Although partisans generally agreed about what hate speech is more deserving of censorship, Republicans and Democrats substantially disagreed on baseline levels of support for censorship. Consistent with *hypothesis 5*, Democrats exhibited significantly more support for censorship of hate speech, regardless of its characteristics. This finding is evident in Fig. 2*A* insofar as Democrats consistently exhibit larger effects compared to the baselines. This pattern becomes even clearer in Fig. 2*B*, which presents marginal means (MMs) of removal and deactivation for each attribute level. This figure shows that partisans substantially differed on the appropriate extent of censorship. Across most attribute levels, Democrats were roughly 10 to 20 percentage points more likely to censor posts. The smallest partisan difference emerged for deactivation when the posts merely contained criticism (4.1 percentage points). The largest partisan difference emerged for removal when the posts contained dehumanizing speech (24.9 percentage points). Democrats were even more likely than Republicans to censor posts targeting Whites, posts from Democrats, and posts from college professors.

### Perceived Censorship Decisions by Political Party.

**Fig. 3.**
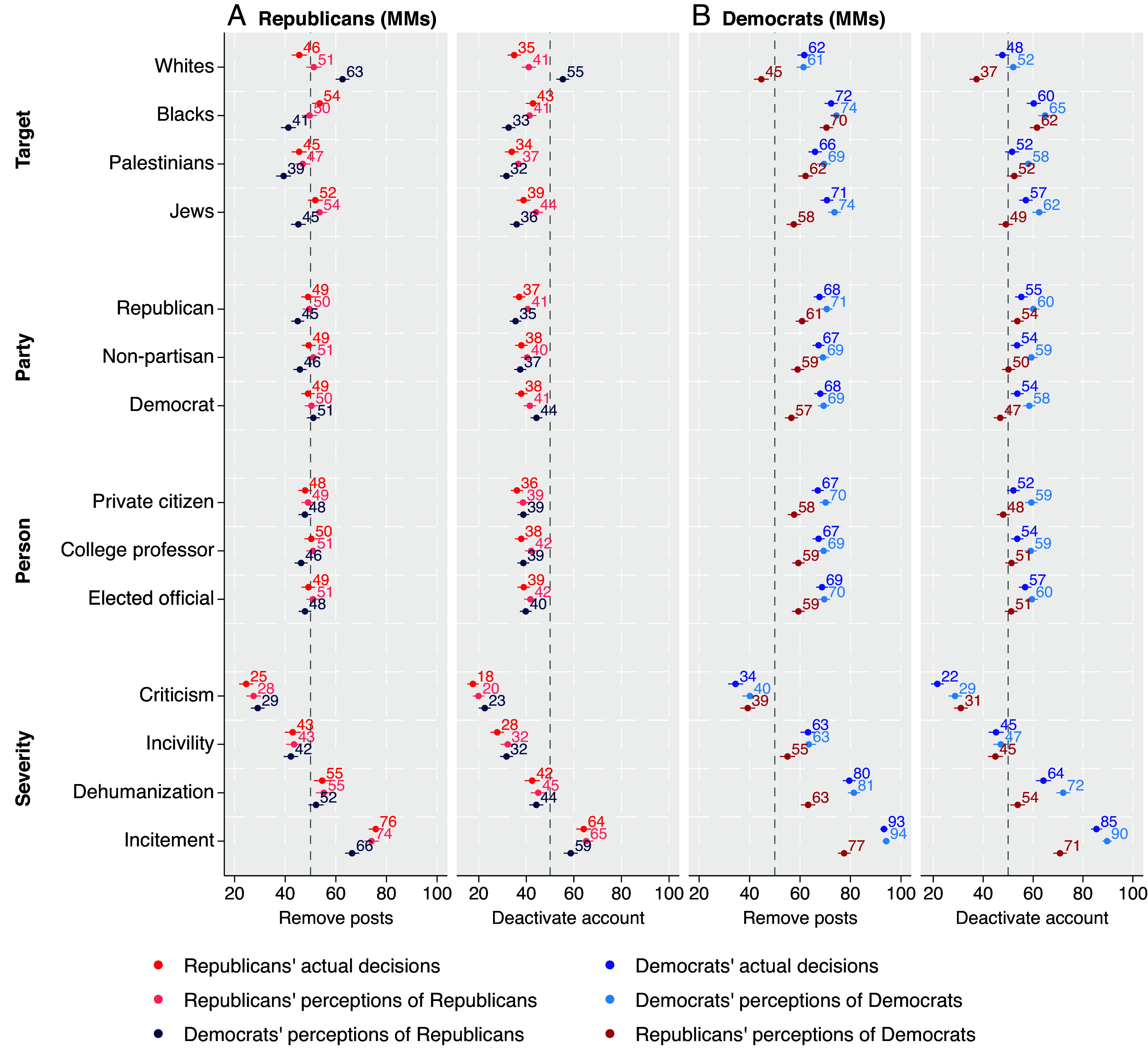
Perceived censorship decisions by target group. Figure reports the MMs of actual decisions, in-party perceptions of decisions, and out-party perceptions of decisions regarding removal and deactivation for (*A*) Republicans and (*B*) Democrats, plotted with 95% CIs. All values are percentage points.

Given this unexpected reality of interparty agreement about what types of hate speech to censor, do partisans actually understand how members of the other party make censorship decisions? Fig. 3 presents the MMs for perceptions of hate speech censorship to facilitate comparisons in absolute terms. (AMCEs are presented in *SI Appendix*.) Fig. 3*A* presents decisions made by Republicans and both Republicans’ and Democrats’ perceptions of how a typical Republican would make those decisions. Recall that our hypotheses about out-party perceptions focused on the targeted group; however, we present the full results with all of the attributes. As predicted by *hypotheses 6* and *7*, Democrats overestimated how often Republicans would censor posts targeting Whites (all *P*’s < 0.01) and underestimated how often Republicans would censor posts targeting Blacks (all *P*’s < 0.01). Also consistent with *hypothesis 7*, Democrats underestimated how often Republicans would remove posts targeting Palestinians and Jews (all *P*’s < 0.01); that said, they did not significantly underestimate how often Republicans would deactivate accounts that posted such content.

Fig. 3*B* presents decisions made by Democrats and both Democrats’ and Republicans’ perceptions of how a typical Democrat would make those decisions. As predicted by *hypothesis 6*, Republicans underestimated how often Democrats would censor posts targeting Whites (all *P*’s < 0.01). However, contrary to *hypothesis 7*, Republicans did not overestimate Democrats’ decisions to censor posts targeting Blacks, Palestinians, or Jews. In fact, Republicans substantially underestimated Democrats’ willingness to censor posts targeting Jews (*P* < 0.01). We suspect that these findings are the result of Democrats’ generally higher support for censorship. That is, Democrats’ baseline support for censorship was so high on average that it was difficult for Republicans to overestimate it in absolute terms.

Overall, even though partisans agree on what to censor, they *perceive* substantial disagreement. Democrats underestimate Republican support for censoring anti-Black, antisemitic, and anti-Palestinian speech. And, although Republicans were fairly accurate in estimating Democratic support for censoring anti-Black and anti-Palestinian speech, they drastically underestimated Democratic support for censoring antisemitic speech. And, quite notably, perceptions of how out-party members will treat anti-White speech is a major disconnect between the two parties-with Democrats overestimating and Republicans underestimating the other party’s willingness to censor speech targeting Whites.

## Discussion

Many debates in contemporary America are experienced through the prism of identity politics. Accordingly, one might think that debates about censoring hate speech stem from partisans’ identification with different targets of prejudice—especially given that hate speech is a prominent manifestation of identity-based social divisions—and, similarly, from partisan differences in assumptions of vulnerability and victimhood (20). Thus, one might expect partisans to be divided about which groups warrant protection from hate speech, as purported by various outlets but not documented empirically (45, 46). However, with the exception of Democrats’ preference for restricting anti-Palestinian speech (relative to anti-White speech), we found no partisan disagreement regarding what types of hate speech to censor-though partisans are unaware of these shared priorities. Partisans mainly disagree about how much hate speech should be censored, with Democrats preferring more than Republicans. Republicans’ prioritization of free speech could be interpreted as complicity with prejudice or a disguise for racism. Indeed, freedom of speech construed as a value can be invoked strategically to justify prejudice, and those with higher levels of racial prejudice are more likely to endorse free speech as a fundamental value in racialized (but not in nonracialized) contexts (13). Even so, the substantial agreement that emerged in our study suggests that Republicans’ disinclination toward censorship should not necessarily be attributed to White identity politics or racism against marginalized groups. And Republicans’ prioritization of free speech appears relatively principled rather than selective. We recognize that the distinction we make may be less relevant from a particular group’s perspective (e.g., the Black community may care about censoring anti-Black speech regardless of whether hate speech targeting other groups is censored). Nonetheless, identifying the basic nature of the partisan disagreement over censorship is essential for developing a civil, just, and free social discourse.

In terms of establishing popular norms of impermissible speech to inform social media policies, it is important to understand that partisans agree on their censorship priorities—despite earnest partisan controversies over cancel culture, so-called “wokeism,” and the Israel–Hamas war. Psychologically, Democrats are associated with Blacks and other marginalized groups and Republicans are associated with Whites and other dominant groups (23)-and partisans, respectively, view these groups as particularly susceptible to harm (19, 20). Yet, we find that partisans on both sides agree that anti-Black and antisemitic speech warrant greater censorship than anti-White speech. Support for restricting anti-Black speech (over anti-White as well as anti-Palestinian speech) seems especially noteworthy given the negative and worsening state of Black versus White race relations in the US (54). Perhaps less surprising is the emergence of bipartisan support for censoring antisemitic speech, as American Jews are a minority group and lean Democrat (55) yet a recent report showed that Republicans perceive greater discrimination against Jews than Muslims, evangelical Christians, and Whites (56). Also, near the time of our data collection, the Republican-controlled House of Representatives passed a resolution denouncing all forms of antisemitism with no mention of anti-Palestinian racism (57). While Democrats support more censorship of anti-Palestinian (versus anti-White) hate speech, partisans on both sides prioritize censorship of speech targeting Blacks and Jews, which could be the result of implicit anti-Palestinian racism (58) or racial gaslighting (59).

Partisan agreement also extended to the null effects of the source’s political party and position in society as well as to the significant effects of the language employed (7, 17). We note that, amid the wide gap in partisans’ attitudes toward academia, Republicans were no more likely to prefer censorship of professors than private citizens (60). An exception to the pattern of partisan agreement is that Democrats appear to hold elected officials to higher standards than private citizens, perhaps as a reaction to Trump’s norm-violating rhetoric (61).

Compared to other studies, we found considerable support for suspending/deactivating accounts (the most severe punishment in this context) across party lines. For example, one recent study found that average support for suspending a social media account that posted threats of violence (the most severe language) targeting LGBTQ individuals was less than 30% (34). (And support for suspension was substantially lower for accounts that threatened violence against Christians, billionaires, and partisans.) In contrast, we found that between 64 and 85 percent of Americans support deactivating accounts that share violent speech targeting racial/ethnic groups, depending on the participant’s partisanship. (See *SI Appendix* for further discussion of recent research on toxic speech versus hate speech in the current study.) Together, these findings reinforce our conclusion that Americans tend to agree on which targets are more versus less deserving of protection from potentially harmful speech (i.e., those whose focal characteristic is ascribed, such as race/ethnicity and sexual orientation, versus not).

Finally, our study shows that partisans mistakenly project identity-based disagreement onto out-partisans. Democrats believe Republicans prefer more censorship of anti-White speech than Republicans actually do. Republicans believe Democrats prefer less censorship of anti-White as well as antisemitic speech than Democrats actually do. These exaggerated partisan stereotypes are particularly noteworthy given the remarkable degree of actual partisan agreement (in terms of what hate speech to prioritize for censorship) in a highly polarized era.

Such misperceptions may stem from narratives propagated by interest groups and the media that perpetuate partisan divisions even when Americans largely agree. For instance, despite the shared belief by 70% of Americans that political correctness is a big problem in the United States, Donald Trump is sometimes depicted as the sole public figure who condemns this phenomenon (43). And although “cancel culture” is deployed across the political spectrum, the media portrays cancellations by liberals versus conservatives in fundamentally different ways, which only emphasizes the partisan cultural divide (62). Partisan misperceptions have important consequences for political communication. These misperceptions could lead members of each party to evade making certain statements or refrain from censoring certain statements due to (an exaggerated) fear of violating a norm held by the other party, akin to a type of pluralistic ignorance. This behavior could have the downstream consequence of partisans opting into communication echo systems because they inaccurately believe there is no agreement on how to assess certain speech. The misperceptions could also contribute to increased distrust and polarization since partisans believe the other side is more divergent from them than they actually are (14).

This research has several limitations. First, we focused on antisemitism and anti-Palestinian speech given the ongoing war (and less attention in academic research) as well as anti-Black and anti-White speech given their significance in American culture. But we did not examine hate speech targeting women, Asian Americans, Latino/Latina Americans, Christians, Muslims, atheists, the LGBTQ community, immigrants, refugees, or other social groups. Future research on hate speech censorship should include additional comparisons. And, although we conducted exploratory analyses examining whether the effects of target group vary by the respondent’s characteristics (*SI Appendix*), it would be informative to examine whether targets of hate speech form differential preferences for censorship. For instance, are certain marginalized groups more or less concerned about regulation of hate speech toward other marginalized groups? And do certain identities (e.g., gender and race) intersect to alter censorship preferences? Also, we noted that our design likely minimized social desirability and/or demand effects, but we cannot entirely rule them out. Future work might inform these possibilities. It would also be interesting to expand the range of sources, including business executives, law enforcement officials, or groups who view themselves as particularly susceptible to cancel culture.

In sum, we find substantial cross-party agreement on what types of hate speech should be prioritized for censorship, yet partisans mistakenly perceive substantial disagreement on this topic. Since the public debate on hate speech moderation often focuses on protecting certain groups at the cost of restricting others, key stakeholders should focus on better understanding what drives misperceptions about censorship, as well as partisan discrepancies in the preferred amount of censorship, rather than what or who should be censored.

## Materials and Methods

We recruited 3,357 participants via Forthright Access between December 8 and December 22, 2023, quota-matched to the US population with regard to age, gender, education, race/ethnicity, region, and partisanship. (Preregistered at https://osf.io/e78ma/?view_only=70c65baad94b4eeab4cbc7ed20a20160.) A power analysis conducted in cjpowR indicated that achieving power of 0.8, with up to four levels per attribute and an effect size of 0.05, requires 6,263 observations or 1,566 participants evaluating four profiles. Therefore, we aimed to recruit a sample of 1,566 Republicans and 1,566 Democrats to facilitate separate analyses for each party. We coded participants who lean toward one party as members of that party. (*N*s = 1,529 Republicans, 1,596 Democrats.) We recruited 216 pure independents for exploratory analyses in *SI Appendix*.

### Attention Check.

Participants were shown a sample profile and then indicated the source’s partisanship and the target group on the next page. Participants who failed this attention check were immediately excluded.

### Demographics and Attitudes.

Before completing the experiment, participants reported demographic information and partisan affiliation, as well as their preference for protecting freedom of expression versus preventing hate speech from spreading (i.e., free speech attitudes).

### Outcomes.

The dependent variables were participants’ decision to remove the posts and/or deactivate the user’s account, as well as their predictions of whether the typical Republican and typical Democrat would remove the posts and/or deactivate the user’s account. Following Kozyreva et al. (3), we dichotomize the deactivation outcome to assess whether participants would deactivate (i.e., temporarily suspend or permanently ban) the account or not (i.e., do nothing or issue a warning).

Each participant evaluated 12 profiles (four personal decisions, four predictions of typical Republicans, and four predictions of typical Democrats), regarding both removal and deactivation for a total of 24 responses (total decisions *N* = 40,284).

### Analyses.

We estimated the causal effects of each attribute on removal and deactivation decisions. We report both AMCEs and MMs (51, 63, 64). AMCEs show effect sizes relative to the chosen reference levels, whereas MMs reflect respondents’ average decisions at each attribute level (64). In *SI Appendix*, we also report a series of exploratory analyses assessing differences by age group, religion, race/ethnicity, and free speech attitudes. We find no notable differences across these groups. We also report separate analyses examining political independents, as well as results using Clayton et al.’s correction for measurement error bias in conjoint survey experiments (65).

### Ethics.

Informed consent was obtained from all participants, and the study was conducted in accordance with relevant guidelines and regulations. The Institutional Review Board at the University of Notre Dame approved the study (protocol #23-11-8187).

## Supplementary Material

Appendix 01 (PDF)

## Data Availability

Data files and scripts necessary to replicate the results are available at https://osf.io/cj5bk/?view_only=d3a332c6707d411293cd9185afe4d0da (66). All other data are included in the manuscript and/or *SI Appendix*.
