# Supplementary material for: Illusory interparty disagreement: Partisans agree on what hate speech to censor but do not know it
Source: Proc Natl Acad Sci U S A. 2024 Sep 16;121(39):e2402428121. doi: 10.1073/pnas.2402428121 (PMC11441537; doi:10.1073/pnas.2402428121)
Supplement: Supplementary file 1 — Appendix 01 (PDF) [file pnas.2402428121.sapp.pdf]

Supporting Information for

Illusory interparty disagreement: Partisans agree on what hate speech to censor but don't know it

This file includes:

1. Sample Demographics
2. Survey
3. Preregistered Research Questions and Hypotheses
4. Average Marginal Component Effects for In- and Out-Party Perceptions
5. Correction for Measurement Error in Conjoint Survey Experiments
6. Deviations from Preregistration
7. Note on Toxic Speech versus Hate Speech
8. SI References

1. Sample Demographics

The final sample included 3,357 participants who evaluated 40,284 profiles. Participants were 46.6% male, 75.4% White, 45.5% Republican, and 47.5% Democratic (counting partisan leaners as partisans), with an average age of 48.4 (*SD* = 17.0). Note that the total sample was skewed toward Whites to ensure a partisan balance. 56.7% of participants prioritized free speech over preventing the spread of hate speech. The educational breakdown was: 22.9% no college, 39.2% some college/vocational training, 26.4% college graduate, 11.5% advanced degree.

2. Survey

[Informed consent]

Generally speaking, do you think of yourself as a …?

- Democrat
- Republican
- Independent
- Other
- Not sure

[If respondent chose Democrat] Would you call yourself a strong Democrat or not very strong Democrat?

- Strong Democrat
- Not very strong Democrat

[If respondent chose Republican] Would you call yourself a strong Republican or not very strong Republican?

- Strong Republican
- Not very strong Republican

[If respondent chose Independent/Other/Not sure] Do you think of yourself as closer to the Democratic or the Republican Party?

- The Democratic Party
- The Republican Party
- Neither
- Not sure

In the next part of the survey, we would like to ask you about your general attitudes towards freedom of expression.

If you absolutely have to choose between protecting freedom of expression and preventing hate speech from spreading, which is more important to you?

- I think protecting freedom of expression is more important than preventing hate speech from spreading.
- I think preventing hate speech from spreading is more important than protecting freedom of expression.

Introduction to the study task

Social media companies sometimes face difficult choices about censoring hate speech. In this study, we are interested in how you would prefer social media sites to approach these difficult choices.

We are going to present you with several scenarios about social media posts and ask you what responses you believe are appropriate, as well as what responses you think certain groups are likely to prefer. You will see 12 scenarios in total. Please assume that the events described in the scenarios are taking place in your country of residence, the United States. This exercise is purely hypothetical. Even if you aren't entirely sure, please indicate which choice seems right to you.

The following table is an example of what these scenarios may look like. **Please carefully read the example table below, and think about whether or not such a post should be removed from the social media site, and whether the account holder should be banned from posting.**

Person	A private citizen
Partisanship	who is a Republican
Target Group	published a series of posts targeting the Jewish community.
Post Content	One of their posts said 'Jews are all just plain stupid.'

What was the partisanship of the person in the prior example? [Wrong answers excluded from the survey]

- Democrat
- Republican
- Non-partisan
- I don't remember

What group did the person target in their social media posts in the prior example? [Wrong answers excluded from the survey]

- Whites
- Palestinians
- Blacks
- Jews

The following tables describe the person making a social media post, their partisanship, the group targeted by the statement, and the statement itself. After examining the table, we will ask you to report what action you would take in response to the post, if you were in charge of the platform.

[Structure of conjoint; not shown to participants]

Attributes	Levels	N of Levels
Person	"An elected official", "A college professor", "A private citizen"	3
Partisanship	"who is a Democrat", "who is	3

	a Republican", "who is non-partisan"	
Target Group	“published a series of posts targeting the Jewish community.”, “published a series of posts targeting the Palestinian community.”, “published a series of posts targeting the Black community.”, “published a series of posts targeting the White community.”	4
Post Content	“One of their posts said ‘[Target group] gets too much attention in our society.’”, “One of their posts said ‘[Target group] are all just plain stupid.’”, “One of their posts said [Target group] aren’t even humans. They’re basically animals.’”, “One of their posts said ‘[Target group] should all be rounded up and shot in the head.’”	4

What would you do with the posts?

- Remove the posts
- Leave the posts online

What would you do with the user’s account?

- Permanently ban their social media account
- Temporarily suspend their social media account
- Issue a warning
- Do nothing

Next, we will present you with another series of scenarios about posts on social media.

This time, we are interested in what you think the **typical [co-partisan]**[1] would prefer to do in different scenarios.

As before, please assume that the events described in the scenarios are taking place in your country of residence, the United States. This exercise is purely hypothetical. Even if you aren't entirely sure, please indicate which choice seems right to you.

The following tables describe the person making a social media post, their partisanship, the group targeted by the statement, and the statement itself. After examining the table, we will ask you to report what action a **typical [co-partisan]** would take in response to the post, if they were in charge of the platform.

Attributes	Levels	N of Levels
Person	"An elected official", "A college professor", "A private citizen"	3
Partisanship	"who is a Democrat", "who is a Republican", "who is non-partisan"	3
Target Group	"published a series of posts targeting the Jewish community.", "published a series of posts targeting the Palestinian community.", "published a series of posts targeting the Black community.", "published a series of posts targeting the White community."	4
Post Content	"One of their posts said '[Target group] gets too much attention in our society.'", "One of their posts said '[Target group] are all just plain stupid.'", "One of their posts said [Target group] aren't even humans. They're basically animals.'",	4

[1] Pure independents were randomly assigned to follow either the Republican or Democratic pathing for the remainder of the survey.

	"One of their posts said '[Target group] should all be rounded up and shot in the head.'"	

What would a **typical [co-partisan]** do with the posts?

- Remove the posts
- Leave the posts online

What would a **typical [co-partisan]** do with the user's account?

- Permanently ban their social media account
- Temporarily suspend their social media account
- Issue a warning
- Do nothing

Finally, we will present you with another series of scenarios about posts on social media.

This time, we are interested in what you think the **typical [out-partisan]** would prefer to do in different scenarios.

As before, please assume that the events described in the scenarios are taking place in your country of residence, the United States. This exercise is purely hypothetical. Even if you aren't entirely sure, please indicate which choice seems right to you.

The following tables describe the person making a social media post, their partisanship, the group targeted by the statement, and the statement itself. After examining the table, we will ask you to report what action a **typical [out-partisan]** would take in response to the post, if they were in charge of the platform.

Attributes	Levels	N of Levels
Person	"An elected official", "A college professor", "A private citizen"	3
Partisanship	"who is a Democrat", "who is a Republican", "who is non-partisan"	3
Target Group	"published a series of posts targeting the Jewish community.", "published a	4

	series of posts targeting the Palestinian community.”, “published a series of posts targeting the Black community.”, “published a series of posts targeting the White community.”	
Post Content	“One of their posts said ‘[Target group] gets too much attention in our society.’”, “One of their posts said ‘[Target group] are all just plain stupid.’”, “One of their posts said [Target group] aren’t even humans. They’re basically animals.’”, “One of their posts said ‘[Target group] should all be rounded up and shot in the head.’”	4

What would a **typical [out-partisan]** do with the posts?

- Remove the posts
- Leave the posts online

What would a **typical [out-partisan]** do with the user’s account?

- Permanently ban their social media account
- Temporarily suspend their social media account
- Issue a warning
- Do nothing

[Link to educational resources regarding hate speech and its consequences]

3. Additional Research Questions and Hypotheses

We preregistered our hypotheses and research questions on the Open Science Framework (available at https://osf.io/e78ma/?view_only=70c65baad94b4eeab4cbc7ed20a20160). We renumbered our hypotheses in the manuscript for presentation purposes. Below is a table of the hypotheses as numbered in the manuscript and in the preregistration.

Hypothesis # in Manuscript	Hypothesis # in Preregistration
Hypothesis 1*	Hypothesis 2(a), 2(b), 2(d)
Corollary 1	Hypothesis 2(c), 2(d)
Hypothesis 2	Hypothesis H4(a)
Hypothesis 3	Hypothesis H4(b)
Hypothesis 4	Hypothesis H3(a), H3(b)
Corollary 2	Hypothesis H3(a)
Hypothesis 5*	Hypothesis 1
Hypothesis 6	Hypothesis 5(b), 5(d)
Hypothesis 7	Hypothesis 5(a), 5(c)

*See Appendix 5 regarding deviations from the preregistration.

Our manuscript focuses on our preregistered main research questions and most of our hypotheses. However, due to space considerations, we did not fully develop the logic behind our severity hypotheses from the preregistration:

- H3a: Republicans are more supportive of censoring hate speech as the severity of the speech increases from criticism to incivility to incitement (but they do not differentiate dehumanization from incivility).
- H3b: Democrats are more supportive of censoring hate speech as the severity of the speech increases from criticism to incivility to dehumanization to incitement.

Around the time of our preregistration, Donald Trump had received substantial media attention for his dehumanizing language regarding immigration and his political opponents, including references to “the communists, Marxists, fascists, and the radical left thugs that live like vermin within the confines of our country” and references to immigration as “a very sad thing for our country; it's poisoning the blood of our country” (1). The prominent and unrepudiated use of this language by the leading presidential candidate for the Republican Party primary suggested that Republicans may be starting to view dehumanizing language as no worse than merely uncivil language. And, a pilot study for this project, conducted on November 24, 2023, supported this theory, finding no difference between Republican support for censoring uncivil and dehumanizing posts. However, contrary to our hypothesis, we found that Republicans (like Democrats) did differentiate between incivility and dehumanization in our main study. We suspect that the results of our pilot study may have been a result of the small sample size or a temporary reaction to the media coverage of Trump’s rhetoric (which had generally receded by the time of our main study in December of 2023).

We also included three exploratory research questions in the preregistration:

- ERQ1: How do the effects of the target group on support for censorship of social media posts containing hate speech vary by age, religion, race, and free speech attitudes?
- ERQ2: How do Democrats' perceptions compare to Republicans' perceptions of Democratic support (Republican support) for censorship of social media posts containing hate speech?
- ERQ3: How do pure independents think social media companies should address posts containing hate speech?

The results addressing ERQ1 are presented in Fig. S1 and Fig. S2. We found no significant differences in the AMCEs of the target group by age group or free speech attitudes. However, we did find a few notable differences regarding religion and race. First, we found that a Black (versus White) target only increased support for censorship among protestants and "none"s (i.e., atheists, agnostics, and those who do not identify with any particular religion). And only the "none"s prioritized censoring anti-Palestinian speech. The AMCEs of antisemitic speech was strongest for Jewish participants. Only Asian American participants did not prioritize censoring anti-Black or antisemitic speech.

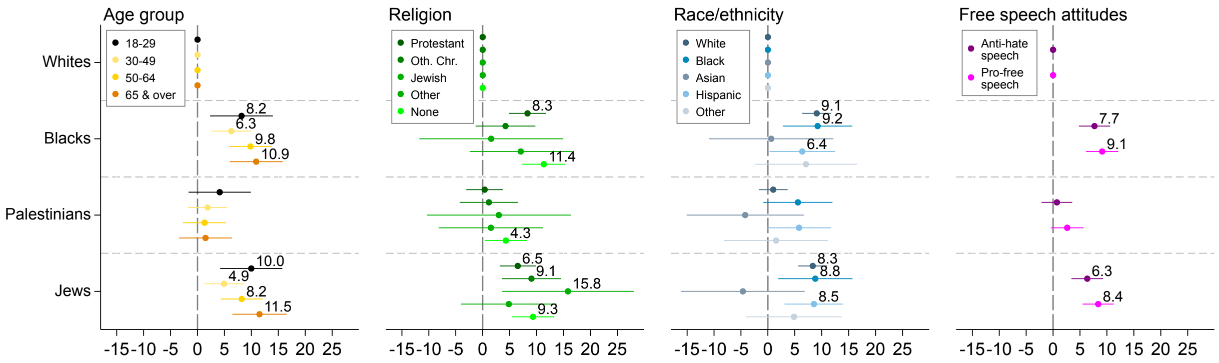

Fig. S1. Target group effects on removal decisions by age, religion, race/ethnicity, and free speech attitudes, plotted with 95% confidence intervals. All values are percentage points.

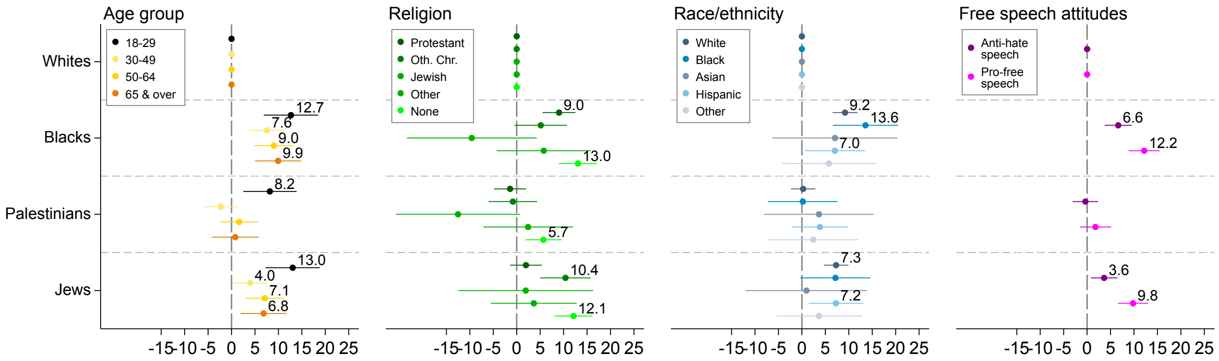

Fig. S2. Target group effects on deactivation decisions by age, religion, race/ethnicity, and free speech attitudes, plotted with 95% confidence intervals. All values are percentage points.

The results addressing ERQ2 are presented in Fig. 3 of the manuscript.

The results addressing ERQ3 are presented in Fig. S3 (pure independents $N = 216$). Regarding AMCEs (Fig. S3A), the effects of anti-Black, anti-Palestinian, and anti-White speech appeared lower for independents than for members of either party. In fact, anti-Palestinian speech had a negative effect on independents' support for deactivation. The effects of hate speech severity on independents were generally in between the effects on Republicans and Democrats. Like Republicans and Democrats, independents were generally not affected by the source of hate speech with one exception: independents were more likely to deactivate accounts owned by elected officials than members of either political party. Regarding MMs (Fig. S3B), independents' censorship preferences were generally between those of Republicans and Democrats, though their preferences tended to be closer to Republicans.

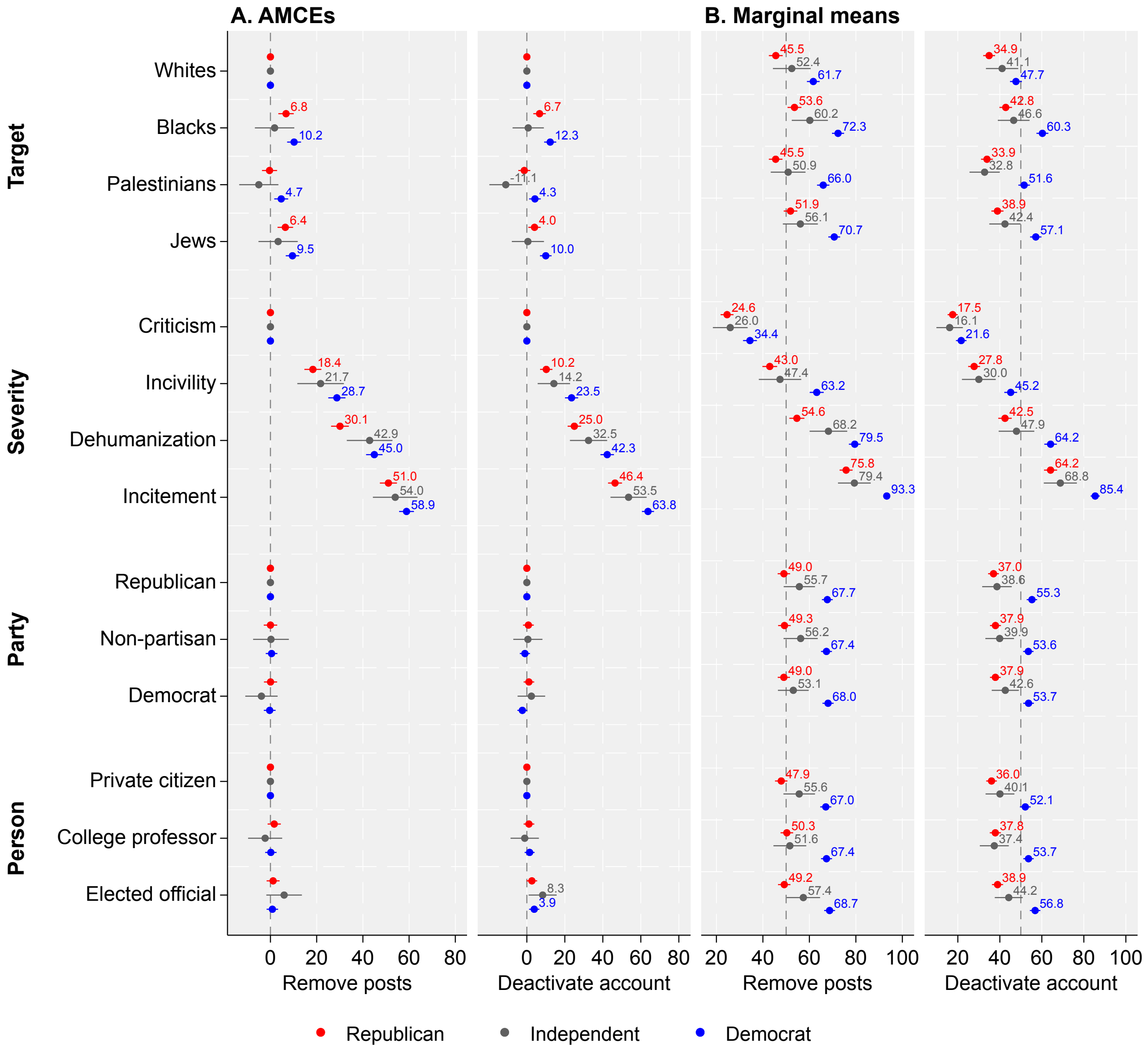

Fig. S3. Figure reports (A) AMCEs and (B) MMs of decisions to remove posts and deactivate accounts by party, including political independents, plotted with 95% confidence intervals. All values are percentage points.

4. Average Marginal Component Effects for In- and Out-Party Perceptions

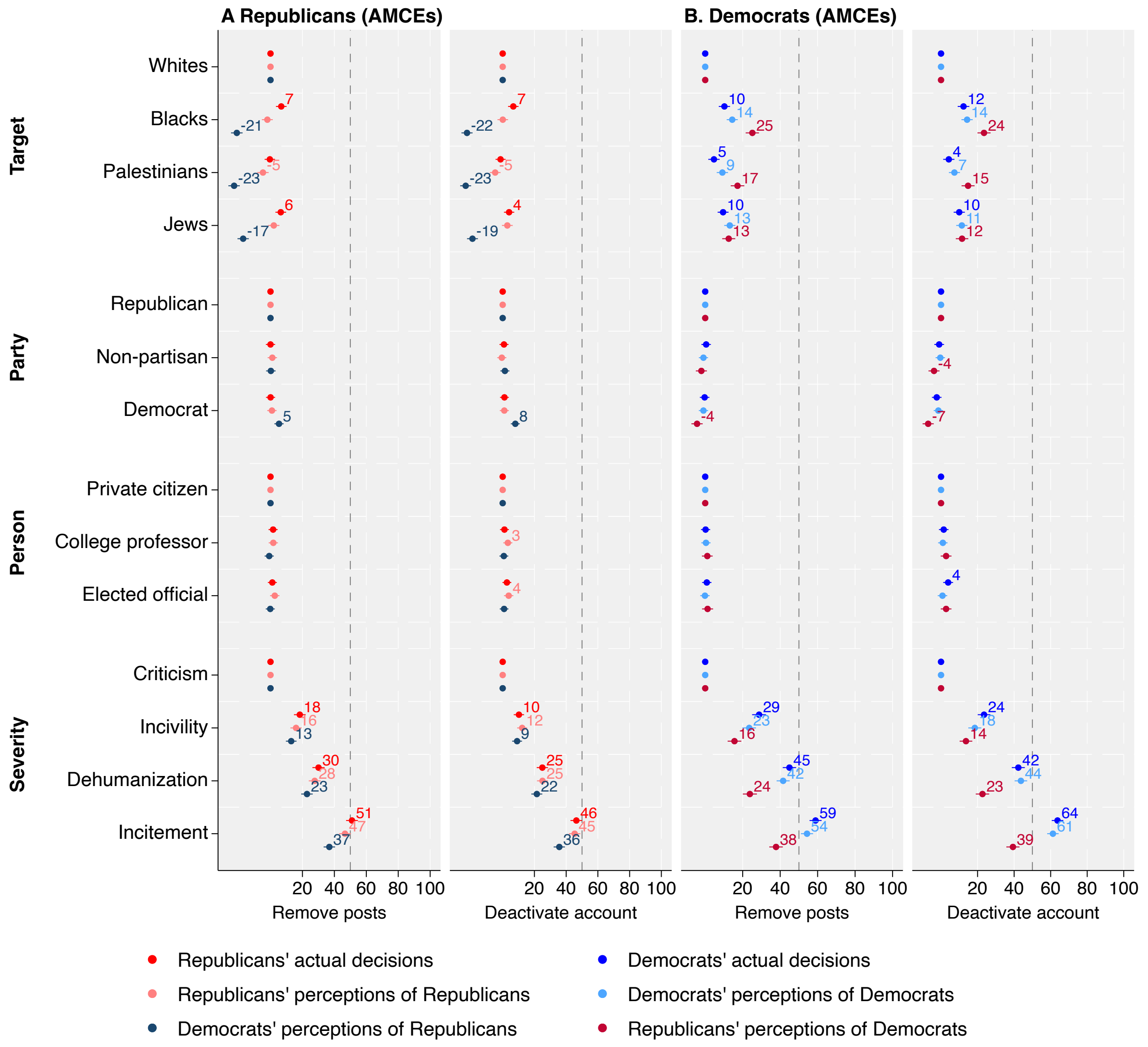

Fig. S4. Figure reports the AMCEs of actual decisions, in-party perceptions of decisions, and out-party perceptions of decisions regarding removal and deactivation for (A) Republicans and (B) Democrats, plotted with 95% confidence intervals. All values are percentage points.

5. Correction for Measurement Error in Conjoint Survey Experiments

Consistent with our preregistration, we explored applying the method for correcting biases induced by measurement error recommended by Clayton et al. (2). This method entails estimating intra-respondent reliability by calculating the percent agreement between the first and last questions and averaging over all respondents or the relevant subgroup. The uncorrected and corrected AMCEs are presented in Fig. S5. The correction did not change any substantive conclusions from our analyses.

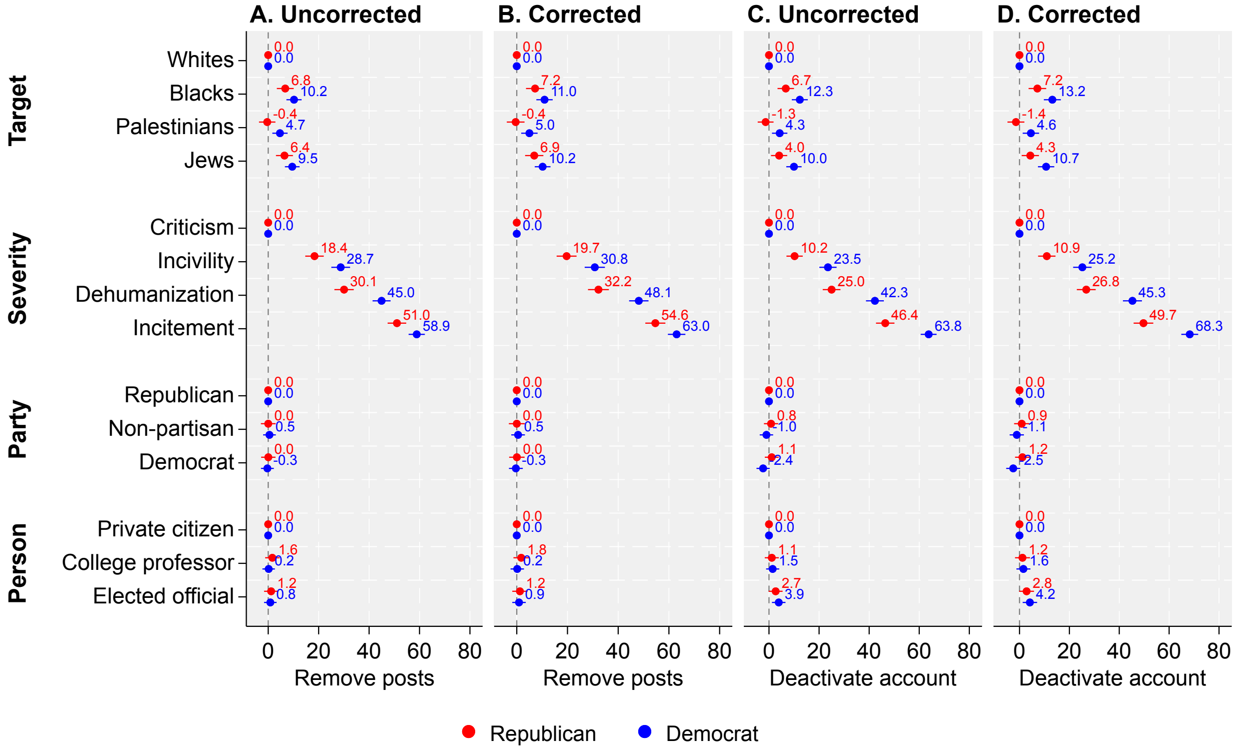

Fig. S5. Figure reports (A) uncorrected and (B) corrected AMCEs of decisions to remove posts and (C) uncorrected and (D) corrected AMCEs of decisions to deactivate accounts by party, plotted with 95% confidence intervals. All values are percentage points.

6. Deviations from Preregistration

Deviation 1: When originally designing this study, we planned to use the same question that Kozyreva and colleagues (3) used to capture free speech attitudes. They used the following item:

> In the next part of the survey, we would like to ask you about your general attitudes towards freedom of expression.
>
> If you absolutely have to choose between protecting freedom of expression and preventing misinformation from spreading, which is more important to you?
>
> - I think protecting freedom of expression is more important than preventing misinformation from spreading.
> - I think preventing misinformation from spreading is more important than protecting freedom of expression.

However, because our study focused on hate speech, we later decided (before the study was preregistered and launched) that it would make more sense to ask about attitudes toward preventing hate speech. Therefore, we actually used this item:

> In the next part of the survey, we would like to ask you about your general attitudes towards freedom of expression.
>
> If you absolutely have to choose between protecting freedom of expression and preventing hate speech from spreading, which is more important to you?
>
> - I think protecting freedom of expression is more important than preventing hate speech from spreading.
> - I think preventing hate speech from spreading is more important than protecting freedom of expression.

We made this change to the actual Qualtrics survey and to the survey instrument that we uploaded as part of our preregistration. However, we failed to change the description of the measure in the measured variables section of our preregistration. Therefore, given the inconsistencies in different parts of our preregistration, we consider this issue a minor deviation from the preregistration.

Deviation 2: In drafting our manuscript, we made a few minor adjustments to our hypotheses that deviate from our preregistration.

First, in the preregistration, we wrote that "(a) Democrats versus Republicans are more supportive of censoring hate speech with three exceptions: (b) speech targeting Whites, (c) speech from Democrats, and (d) speech from college professors." However, we did not find those exceptions. Therefore, rather than attempting to build up the logic for those exceptions that we did not find, we simplified the hypothesis to simply: "relative to Republicans, Democrats may exhibit more support for censorship of hate speech, all else constant (*hypothesis 5).*"

Second, in the preregistration, we failed to specify that we expected Republicans to be more supportive of censoring anti-White speech versus anti-Palestinian speech. This was an oversight on our part; we intended to include that hypothesis. However, we did not actually find that pattern in the data. Therefore, our hypotheses were consistent with our findings, but only because of an oversight on our part.

7. Note on Toxic Speech versus Hate Speech

Here, we briefly address some aspects of (dis)similarity between our work and Pradel and colleagues (4), which focused on censorship of toxic speech. First, Pradel and colleagues found that, with the exception of their experiment in which LGBTQ individuals were targeted, the majority of respondents did not support any content moderation. However, the lack of such support pertains to their experiments in which the targeted groups were Christians and billionaires (which enjoy majority and privileged statuses, respectively, in society) and, with the exception of threatening posts, Republicans and Democrats (which enjoy equal status in society). In our study, we found that the majority of respondents *did* support removal. But, of course, we focused on race/ethnicity (Blacks, Whites, Jews, and Palestinians). Thus, the ascribed nature of the targeted groups' focal characteristic is a key element that differed across Pradel et al. (with the exception of LGBTQ targets) and our work. We note that, while Jews and Christians are both religious targets and one can choose their religious beliefs, Jews (unlike Christians) are minorities that have a long history of facing ethnic-based discrimination (e.g., the Nazis disregarded whether Jews were religiously observant or not). We also note that the majority of respondents in our study supported the removal of posts that targeted Whites. Although Whites are a social majority group, this pattern suggests that race/ethnic-based hate speech is less tolerated than toxic speech targeting groups whose focal attribute is more controllable (i.e., being a Christian, billionaire, Republican, or Democrat). Thus, from our view, our findings are distinct from, rather than inconsistent with, Pradel and colleagues. That said, like Pradel et al., we found that Democrats were more apt to support content moderation than Republicans (and the partisan differences in effects of the targeted group were usually in terms of magnitude rather than whether or not there was a significant effect).

We also acknowledge that Pradel et al.'s operationalization of toxic speech is similar to how we operationalize hate speech. For instance, Pradel et al. conclude that incivility, intolerance, and violent threats should be thought of as distinct types of toxicity (rather than a continuum), though uncivil and intolerant content elicited similar responses (such that the majority of respondents did not support removal of either type of content). And they found that violent threats are more likely to prompt support for removal, depending on the targeted group. We found that the majority of our participants did not support removal of criticism and Republicans did not support the removal of incivility, but Democrats supported the removal of incivility and that the majority of respondents in both parties supported removal of dehumanization and incitement (and such findings did seem to operate as a continuum, increasing in severity). Pradel et al. note that, "other manifestations of intolerance… may be relevant and future work should consider treatments that move beyond ours that expose respondents to "language whose scope is discriminatory and/or exclusionary and/or derogatory," which they suggest may elicit different responses. And, indeed, the condition closest to this that we examined (i.e., dehumanization) as well as incitement (similar to violent threats) did prompt greater support for content moderation. Thus, our findings are not wholly inconsistent with Pradel et al., and both narratives should be retained, keeping in mind the targets of the social media posts. Indeed, differences in effects for incivility may be due to differences in targeted groups, such that the ascribed nature of the group's focal characteristic matters.

8. SI References

1. Kurtzleben, D. Why Trump's authoritarian language about 'vermin' matters (2023) https://www.npr.org/2023/11/17/1213746885/trump-vermin-hitler-immigration-authoritarian-republican-primary/.
2. Clayton, K., Horiuchi, Y., Kaufman, A.R., King, G., & Komisarchik, M. Correcting measurement error bias in conjoint survey experiments (N.D.) Working Paper. http://tinyurl.com/24btw3dq.
3. Kozyreva, A., Herzog, S. M., Lewandowsky, S., Hertwig, R., Lorenz-Spreen, P., Leiser, M., & Reifler, J. (2023). Resolving content moderation dilemmas between free speech and harmful misinformation. *Proceedings of the National Academy of Sciences*, *120*(7), e2210666120. https://doi.org/10.1073/pnas.2210666120.
4. Pradel, F., Zilinsky, J., Kosmidis, S., & Theocharis, Y. (2024). Toxic Speech and Limited Demand for Content Moderation on Social Media. *American Political Science Review*, 1-18. https://doi.org/10.1017/S000305542300134X.
